# ﻿Lectotypification of *Salvia bogotensis* Benth. (Lamiaceae)

**DOI:** 10.3897/phytokeys.265.165733

**Published:** 2025-10-14

**Authors:** Ángela María Morales-Trujillo, Brenda Yudith Bedolla-García, Patricia Hernández-Ledesma, Jorge Gabriel Sánchez Ken

**Affiliations:** 1 Instituto de Ecología A.C. (INECOL), Centro Regional del Bajío, Red de Diversidad Biológica del Occidente Mexicano, Avenida Lázaro Cárdenas 253, 61600 Pátzcuaro, Michoacán, Mexico Centro Regional del Bajío, Red de Diversidad Biológica del Occidente Mexicano Pátzcuaro Mexico

**Keywords:** *
Calosphace
*, Colombia, herbaria

## Abstract

The species *Salvia bogotensis* (sect. Angulatae) was not adequately typified at the time of its description. The original material used by the author of the species corresponds to collections by *Goudot s.n.* carried out in Bogotá, Colombia. There are five specimens that match with this data distributed in three herbaria: F, K and P. Epling designated the material deposited in K as lectotype, however, these specimens rather correspond to two different collections, and he failed to choose one of them unambiguously. Because of this, it is necessary to clearly determine which of the two specimens corresponds to the lectotype. The specimen K000479515 (K!) is designated here as lectotype because it is the most complete showing the key morphological characters of the species (leaves, inflorescences, and flowers), in addition to the note made by Epling. Of the other specimens, one of those deposited at P(P00715001) is an isolectotype.

## ﻿Introduction

The sixth-largest angiosperm family is LamiaceaeMartinov (1820: 355). It has a cosmopolitan distribution and contains about 236 genera and 7200 species (Kubitzki 2004). *Salvia* L. (In Linnaeus 1753: 23) is the largest genus of Lamiaceae, comprising approximately 1000 species, and also has a cosmopolitan distribution (Drew et al. 2017; Drew 2020). It occurs in diverse biomes from sea level to 4000 m elevation (Claßen-Bockhoff et al. 2004; Kriebel et al. 2019), with centers of diversity in Eurasia, including the Mediterranean (ca. 250 spp.) and Central and Eastern Asia (ca. 90 spp.), as well as Eastern and Southern Africa (ca. 60 spp.), North America including Mexico (ca. 314 spp.), Central America (ca. 164 spp.), and South America (ca. 330 spp.) (Claßen-Bockhoff et al. 2004; Walker et al. 2004; Kriebel et al. 2019; González-Gallegos et al. 2020).

The genus *Salvia* is classified worldwide into 11 subgenera, two of which are endemic in the American Continent: *Audibertia* J. B. Walker, B. T. Drew, & K. J. Sytsma (2015: 837) is restricted to the United States and northern Mexico and *Calosphace* (Benth.) Epling (Bentham 1833: 198; Epling 1939: 4) with approximately 582 species, has about half of the species richness of the genus, and it is distributed from the southern United States, to Argentina, including the Caribbean islands, covering a total of 42 countries (González-Gallegos et al. 2020). Mexico, the Andes, southern Brazil and Argentina are a center of diversity of *Calosphace* (Walker and Sytsma 2006). Mexico is the primary center of diversification of the subgenus with 295 spp., of which 243 are endemic. This places the country as the richest, followed by Peru with 77 spp. (49 endemics) and Colombia with 60 spp. (37 endemics) (González-Gallegos et al. 2020).

Based on floral morphology and geographic distribution, the subgenus Calosphace is divided (Walker et al. 2004) into 104 sections (Epling 1939; Epling and Mathias 1957). One of these sections, *Angulatae* (Epling 1939: 234), represents a major challenge due to its species richness (52) (Epling, 1937, 1939, 1940, 1941; Epling and Mathias 1957; Epling and Játiva 1966) and wide geographic range, extending from the southern United States to Argentina and the Caribbean islands (González-Gallegos et al. 2020). However, recent phylogenetic studies (Fragoso-Martínez et al. 2018) have confirmed that section Angulatae is polyphyletic, with species distributed across at least eight distinct clades.

In Colombia, section Angulatae is represented by 14 species, primarily distributed in Cordillera Oriental (Boyacá and Cundinamarca departments) and Sierra Nevada de Santa Marta (Magdalena department), where several microendemisms occur. Wood and Harley (1989) included the section in their taxonomic revision of *Salvia* for Colombia, while Fernández-Alonso (2003) provided a synopsis of the Colombian species in the section; both works included *Salvia bogotensis* Benth. (1848: 312). After reviewing the nomenclature of this species, we found that the information regarding the type specimen is ambiguous, as it is only noted that the type is at K, where there are two specimens, in addition to three more in other herbaria. This study arises from the need to clarify the typification of this species as an essential step in the taxonomic research of the genus.

## ﻿Materials and methods

All information regarding *Salvia bogotensis* was gathered from Tropicos.org (https://www.tropicos.org/home), and the protologue was consulted in the Botanicus Digital Library (2024) (http://www.botanicus.org/). Other online resources such as type images in JSTOR Global Plants 2025 (https://plants.jstor.org/), the Mid-Atlantic Herbaria Consortium (https://midatlanticherbaria.org/), and the Kew Data Portal (2025) (https://species.data.kew.org/) were examined. Typification of names follows the International Code of Nomenclature for Algae, Fungi, and Plants (ICN) (Turland et al. 2018). Herbarium acronyms are according to Thiers (2017 onwards).

## ﻿Typification of the name

*Salvia bogotensis* was described by the English botanist George Bentham in 1848. The specific epithet refers to the collection locality in Bogotá, Cundinamarca, Colombia, an area extensively explored in the early 1800s by the French naturalist *Justin Goudot* (JSTOR 2025). In the protologue, Bentham (In De Candolle 1848: 312) states: “*prope* Bogotá, *Goudot*! (v. in h. Hook.)”, indicating that he examined the specimens of the Hooker’s herbarium that was incorporated at Kew Herbarium, where Bentham worked and where most of his type specimens are deposited (O’Leary and Moroni 2016).

**Figure 1.**
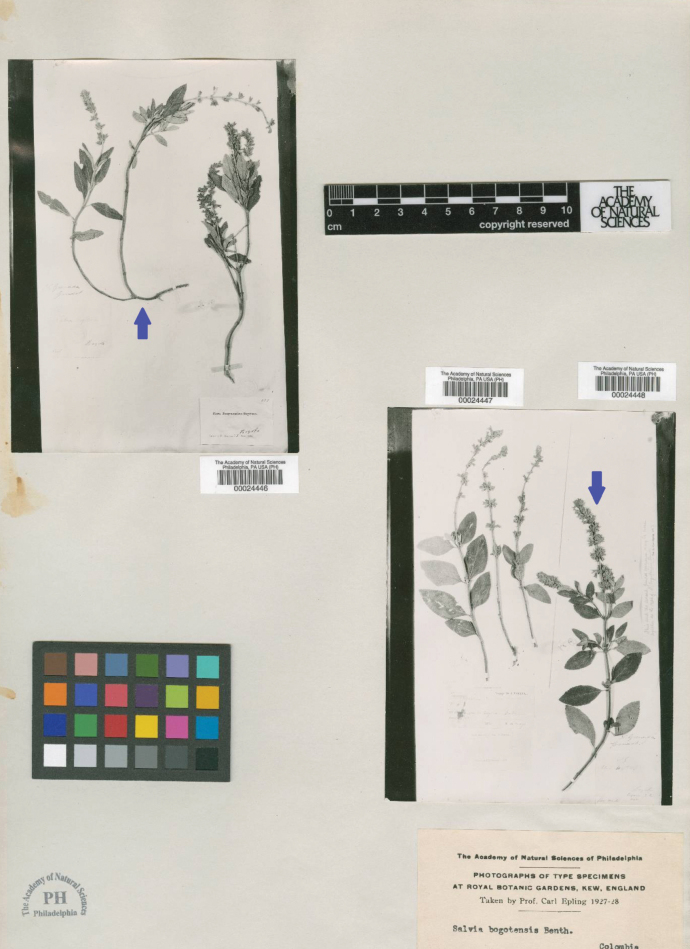
Photographs of a mixed collection of *Salvia bogotensis* Benth. (Lamiaceae) a photo of “type” from the Academy of Natural Sciences [PH (PH00024448, PH00024446, PH00024447)]. The arrows refer to the collections of *Goudot s.n*.

Two *>Goudot’s* specimens with original labels were deposited at K, K000479514, collected in “Bogotá” in April (as avril), and K000479515, in “Bogotá” locality of “Boquerón” in March (as *martii* (Latin)) both correspond to “*Nelle. [Nouvelle] Grenade*”. At the time of collection, Colombian territory was officially known as the Republic of New Granada, a name written on the labels. The locality “Boquerón” likely refers to the páramo of the same name surrounding Bogotá. Epling (1939), in his monograph on American Salvias, only mentioned that he saw the type of *S. bogotensis* at K (*Goudot’s* collection). However, upon reviewing the online specimens, we found that there are currently five sheets and a photo of a “type”: two in K, two in P, one in F and another in PH (photo). Epling left a note on the specimen K000479515 in 1927 stating: ‘This and the second *Goudot* specimen may be taken together as the type.’ However, in Kew both correspond to different specimens collected at different times and places K000479514 and K000479515. Meanwhile, a photo of a “type” (PH00024448, Fig. 1) was also located at PH (Thiers 2017), containing photographs taken by Epling between 1927 and 1928 of the specimens from K, along with images of specimens collected at the type locality by *Isaac F. Holton 479* (23 November 1852, PH00024446) and *J. Triana 3597* (11 May 1855, PH00024447), both collected at the same location. Additionally, other *Goudot’s* specimens collected in Bogotá were located: two in the P herbarium, P00715001 collected in Bogotá, locality of Boquerón, in March of 1844. This specimen is a duplicate of K000479515. And the specimen P00715002 collected in Bogotá in April of 1844 is a duplicate of K000479514. Finally, one in F (F0061175F - Catalog Number: 976885), without collection date.

**Figure 2.**
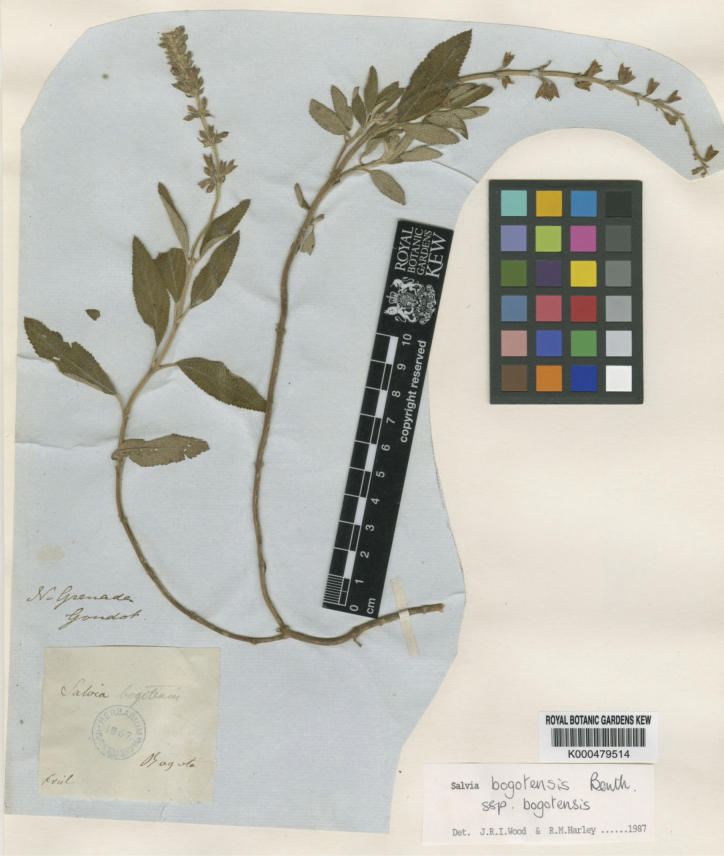
Specimen of *Salvia bogotensis* Benth. (Lamiaceae) *Goudot s.n.* (K000479514), collected in April.

**Figure 3.**
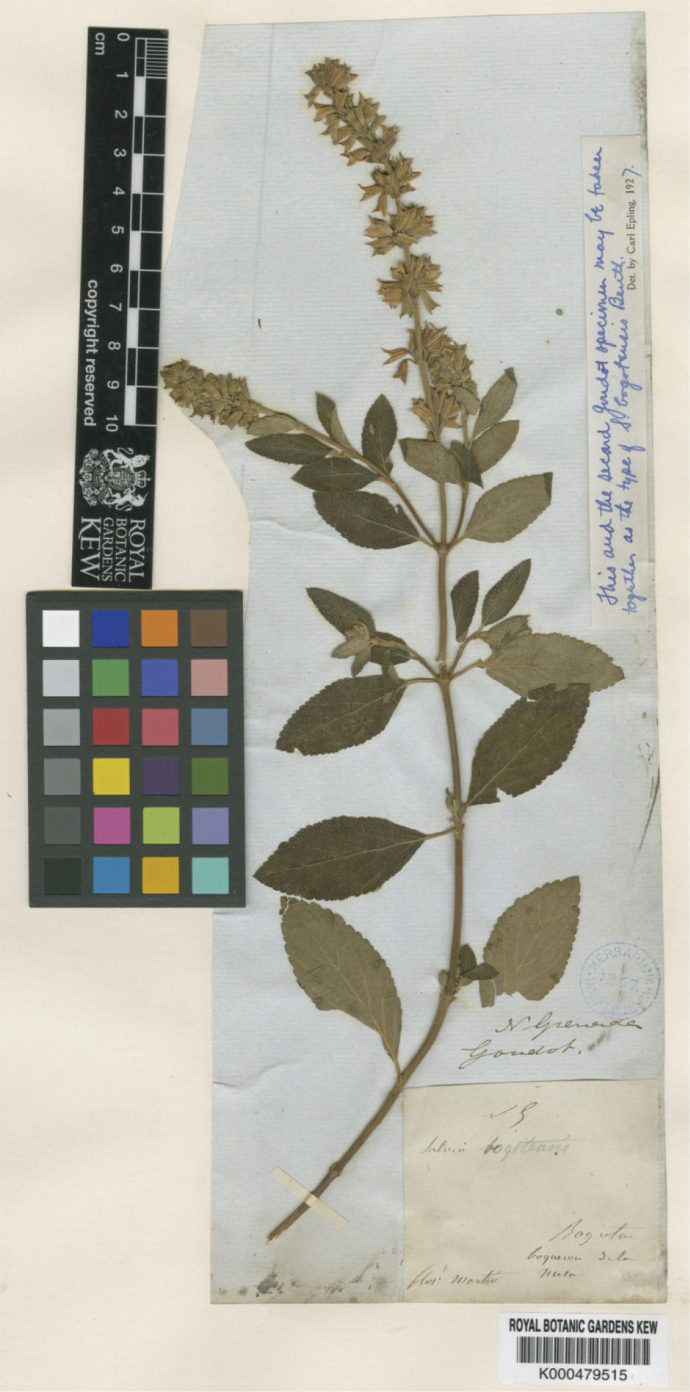
Lectotype of *Salvia bogotensis* Benth. (Lamiaceae) *Goudot s.n.* (K000479515), collected in March.

The photos of types reveal that the Kew specimens were originally mounted onto two mixed sheets: the first included a *Goudot* specimen alongside one collected by Holton, while the second combined a *Goudot* specimen with one collected by *Triana*. This means that both K sheets contained four different collections that later were separated and are now cataloged as individual specimens: *Goudot’s* (K000479514, Fig. 2; K000479515, Fig. 3), *Holton’s* (K004937578), and *Triana’s* (K004937579). Mixed-sheet mounting was a common practice implemented in the 1930s during the Great Depression (Forman and Bridson 1989). This practice was due to limited resources during the 19^th^ and early 20^th^ century, as mounting materials were costly and scarce. This forced institutions and collectors to group multiple specimens on the same sheet to save costs and storage space; however, there were misidentification errors, where collectors inadvertently grouped different species under the same collection number, a situation detected later during taxonomic revisions (Forman and Bridson 1989).

By leaving a note on the specimen, Epling indicated that the material from the two sheets at K should be together as the type; they belong to two different collections and therefore cannot be considered one type specimen. For this reason, it is likely that the K staff mounted each plant separately without cross-referencing labels indicating they constituted a single specimen (ICN, Art. 8.3). Epling later reviewed additional type materials of *Salvia bogotensis* in other herbaria, wrongly designating P00715001 and P00715002 (P) as isotypes in 1928, and possibly F0061175F (F) as an isotype, though the authorship of this annotation is unclear. Regardless, duplicates of this collection have been found in multiple herbaria, all considered part of the same original collection (ICN, Art. 8.2).

**Figure 4.**
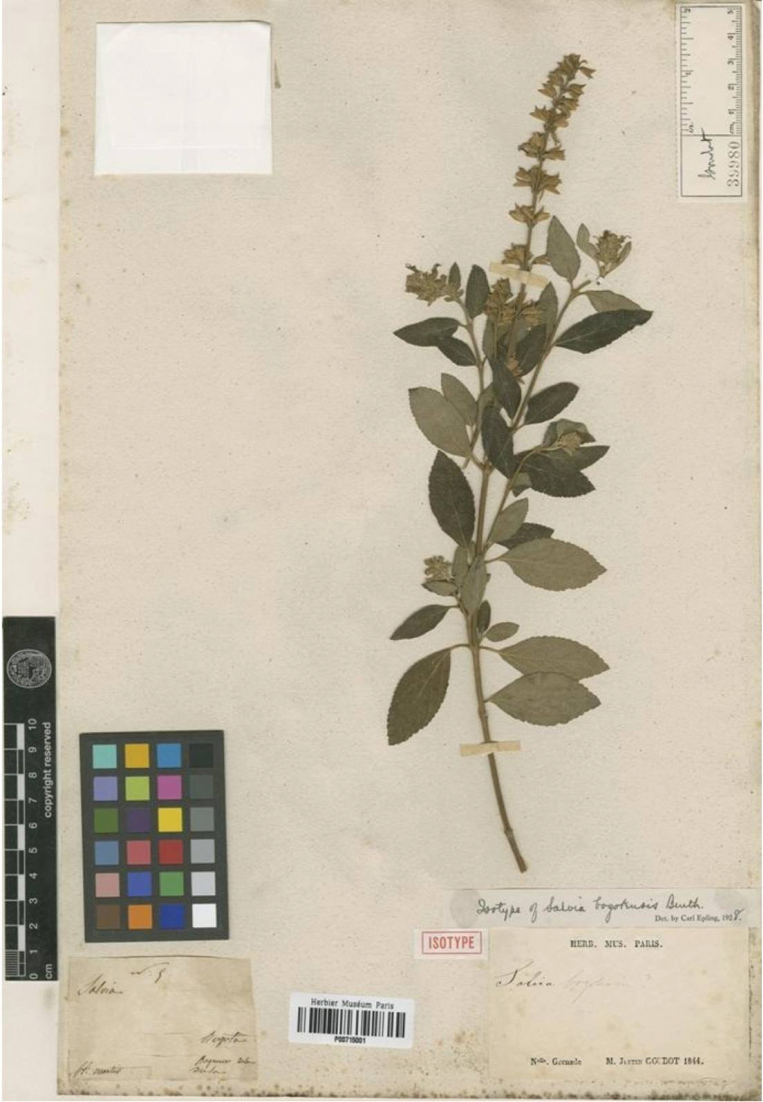
Isolectotype of *Salvia bogotensis* Benth. (Lamiaceae) *Goudot s.n.* (P00715001), collected in March.

After the name *Salvia bogotensis* was published, in all subsequent studies the specimens deposited at K were considered the type collection (Wood and Harley 1989; Fernández-Alonso 2003), without mentioning clearly which of the two specimen represents the type. Following the ICN Arts. 9.3, 9.14, and 9.17, a lectotype must be designated from the original material. If the lectotype designation refers to a single gathering but multiple specimens, a second-step lectotypification must restrict it to one specimen. Therefore, here we designate K000479515 as the lectotype that has a more complete specimen (leaves, inflorescences, and flowers) and its inclusion of an original note by Epling supporting its selection. Only one of the others *Goudot’s* collection, those of P (P00715001) correspond to an isolectotype (Fig. 4) since it has the same collection date of the lectotype.

### ﻿ Salvia bogotensis

Taxon classificationPlantaeLamialesLamiaceae

Benth., Prodr. [A. P. De Candolle] 12: 312. 1848.

2A776169-21B8-5A70-8458-892F9A85D78F

#### Lectotype.

Colombia • Dep. Cundinamarca, Boquerón, Bogotá, N. [*Nouvelle*] Granade, *M. J. Goudot s.n.* 1844 (K! first-step designed by Epling 1939:254), Second-step designated here: — K [barcode K000479515 image!]; isolectotype: P [barcode P00715001 image!].

#### Note.

In a handwritten annotation on specimen K000479515 (*Goudot s.n*.), in 1927, Epling stated that this specimen should together be taken with K000479514 to jointly constitute type material. However, this last one corresponds to another collection with another date and locality collection.

##### ﻿Further remarks

*Salvia bogotensis* is an endemic species of Colombia that grows in high-altitude regions (2500–4000 m) in El Cocuy (Boyacá), located at the Northern end of the Eastern Mountain range. It is characterized as a small to medium-sized shrub with narrow, bullate leaves, moderately short inflorescences, and an intense blue corolla (Fernández-Alonso 2003).

On the other hand, Wood and Harley (1989) recognized three subspecies of *Salvia bogotensis* Benth. complex: subsp. aratocensis, subsp. sochensis, and subsp. bogotensis, which exhibit overlapping distributions in Boyacá and Santander. Fernández-Alonso (2003) later elevated the first two to species rank (*Salvia aratocensis* and *S. sochensis*), while reclassifying S. bogotensis subsp. suratensis as S. aratocensis subsp. suratensis due to its close morphological affinity and restricted distribution to the Chicamocha Valley (Santander, department) (Fernández-Alonso 2003).

## Supplementary Material

XML Treatment for Salvia bogotensis

## ﻿Additional information

### Use of AI

No use of AI was reported.

### Author ORCIDs

Ángela María Morales-Trujillo https://orcid.org/0000-0002-5723-0160

Brenda Yudith Bedolla-García https://orcid.org/0000-0002-1560-1090

Patricia Hernández-Ledesma https://orcid.org/0000-0002-0071-4201

Jorge Gabriel Sánchez Ken https://orcid.org/0000-0001-9623-7245

### Data availability

All of the data that support the findings of this study are available in the main text.
